# Empirically derived dietary patterns in relation to periodontitis and number of teeth among Norwegian adults

**DOI:** 10.1017/S1368980023002690

**Published:** 2024-01-15

**Authors:** Natalia Petrenya, Magritt Brustad, Laila A Hopstok, Gro Eirin Holde, Birgitta Jönsson

**Affiliations:** 1 The Public Dental Health Service Competence Centre of Northern Norway, P.O Box 2406, N-9271, Tromsø, Norway; 2 Department of Community Medicine, Faculty of Health Sciences, UiT The Arctic University of Norway, Tromsø, Norway; 3 Department of Health and Care Sciences, Faculty of Health Sciences, UiT The Arctic University of Norway, Tromsø, Norway; 4 Department of Clinical Dentistry, Faculty of Health Sciences, UiT The Arctic University of Norway, Tromsø, Norway; 5 Department of Periodontology, Institute of Odontology, The Sahlgrenska Academy, University of Gothenburg, Gothenburg, Sweden

**Keywords:** Periodontitis, Number of teeth, Alveolar bone loss, Dietary patterns, Principal Component Analysis

## Abstract

**Objectives:**

To explore dietary patterns in relation to periodontitis and number of teeth.

**Design:**

A cross-sectional study.

**Setting:**

We used data from the seventh survey of the Tromsø Study in Norway, 2015–2016. Three periodontitis groups were compared: (i) no periodontitis/slow bone loss; (ii) moderate bone loss; and (iii) rapid bone loss. Number of teeth was categorised as 25–28, 20–24 and ≤ 19. Dietary patterns were identified by principal component analysis. Multiple logistic regression was applied to examine associations between tertiles of dietary pattern scores and periodontitis, and between these same tertiles and number of teeth.

**Participants:**

1487 participants (55·5 % women) aged 40–79 years who were free of major chronic diseases, attended an oral health examination and completed a FFQ.

**Results:**

Four dietary patterns were identified, which explained 24 % of the total variability in food intake: fruit and vegetables, Westernised, meat/fish and potatoes, and refined grain and dessert. The fruit and vegetables pattern was inversely associated with periodontitis characterised by rapid bone loss when compared with no periodontitis/slow bone loss (OR tertile 3 *v.* 1 0·49, 95 % CI: 0·25, 0·98). Participants who were in the highest tertile of the refined grain and dessert pattern (tertile 3 *v.* 1) had 2·38- and 3·52-fold increased odds of having ≤ 19 than 20–24 and 25–28 teeth, respectively.

**Conclusion:**

Out of four identified dietary patterns, only the fruit and vegetables pattern was negatively associated with advanced periodontitis. A more apparent positive association was observed between the refined grain and dessert pattern and having fewer teeth (≤ nineteen teeth).

Periodontitis remains a highly prevalent dental disease worldwide despite general improvements in oral hygiene, such as toothbrushing and interproximal cleaning, and availability of oral health services^(1)^. Central to periodontitis is chronic inflammation and progressive destruction of the supporting tissues of the teeth as an excessive immune response to specific bacterial colonisation of dental plaque.

Periodontitis shares numerous risk factors with systemic, chronic diseases like cardiovascular diseases and diabetes, e.g. age, smoking, unhealthy diet, stress and hormonal changes^(2)^. Preventive measures against periodontitis should comprise periodontal infection control, i.e. gingivitis management^(3)^, and the promotion of healthy lifestyle behaviours. It has been suggested that a healthy, balanced diet can help reduce the risk of periodontitis; recent evidence has highlighted associations between periodontitis and micronutrient deficiencies, high consumption of saturated fats and fermentable carbohydrates^(2,4)^.

Recently, several studies have attempted to assess the relationship between overall diet and periodontitis^(5–11)^. Most studies have used hypothesis-driven dietary patterns based on *a priori* indices, such as indices derived using intake of foods and nutrients correlated with inflammatory biomarkers, e.g. the Dietary Inflammatory Index (mainly nutrient-based)^(6,7)^ and anti-inflammatory dietary score (based on nine food groups)^(11)^, as well as indices that measure adherence to established evidence-based dietary patterns for chronic disease prevention, e.g. the Dietary Approaches to Stop Hypertension and the Mediterranean Diet Score^(9,12)^, plant-based diet indices^(13)^ and food groups according to the degree of processing^(14)^. Most studies of *a priori* indices reported lower adherence to the anti-inflammatory/healthier diet in individuals with periodontitis^(6,7,9,11,13,14)^. However, hypothesis-driven approaches neither reflect overall dietary patterns nor consider the correlated structure of the dietary components and nutrients^(15)^. Commonly, *a priori* indices are not developed specifically for the target population; thus, they may not fully reflect the dietary behaviour of this group. Even though data-driven or *a posteriori* dietary patterns have limited generalisability, empirically derived dietary patterns provide important knowledge that complements the findings of hypothesis-driven methods^(16)^. To date, few population-based epidemiological studies on the association between overall diet and periodontitis have focused on *a posteriori* dietary patterns on the basis of variation in food group intake^(5,8,10)^ and those that do exist rendered inconsistent results. A longitudinal study found no overall association between the Westernised and Prudent dietary patterns, determined by principal component analysis, and self-reported periodontitis^(5)^. A cross-sectional study reported that a dietary pattern rich in salad, fruit and vegetables, poultry, seafood, and plain water or tea, as identified by treelet transformation, was associated with a lower extent of objectively measured periodontitis (i.e. proportion of sites with clinical attachment loss (≥ 3 mm)^(8)^. Further, it has been suggested that obesity could be an effect modifier in the positive association between the Western dietary pattern and periodontitis, as the association has been found to be significant only in individuals with obesity^(5)^. Another study found no overall association between a pro-inflammatory diet and the risk of self-reported periodontitis, except among non-smokers with obesity, using reduced rank regression, which is an *a posteriori* method but incorporates prior knowledge about diseases and their pathways^(10)^.

Functional dentition (i.e. having ≥ 20 natural teeth) is important for chewing, speech and dental aesthetics. It is well known that periodontitis contributes to extensive or even complete tooth loss, especially among older adults. An association between an anti-inflammatory diet, investigated using the Dietary Inflammatory Index, and fewer missing teeth has recently been demonstrated^(17)^. It is, however, unclear whether empirically derived dietary patterns are associated with extensive tooth loss. Thus, more research is needed to identify which dietary patterns are associated with periodontitis and tooth loss.

The aim of the present study was to explore empirically derived dietary patterns in relation to periodontitis and number of teeth in a general Norwegian population.

## Materials and methods

### Study population

The Tromsø Study is an ongoing population-based study in Tromsø, Norway. Seven surveys (Tromsø1-Tromsø7) have been conducted between 1974 and 2016, to which total birth cohorts and random population samples have been invited (attendance 65–79 %)^(18)^. Data collection methods comprise questionnaires and interviews, biological sampling and clinical examinations.

### Study sample

The present study includes participants from the seventh survey of the Tromsø Study 2015–2016 (Tromsø7)^(19)^. All inhabitants aged ≥ 40 years were invited (*n* 32 591), and 65 % attended (*n* 21 083, aged 40–99 years, 53 % women). Of these, a random subsample of 3943 participants attended an oral health examination. Several questionnaires were completed including a thirteen-page semi-quantitative FFQ^(20)^, developed and validated at the University of Oslo^(21)^, distributed to the participants at the examination site.

**Fig. 1:**
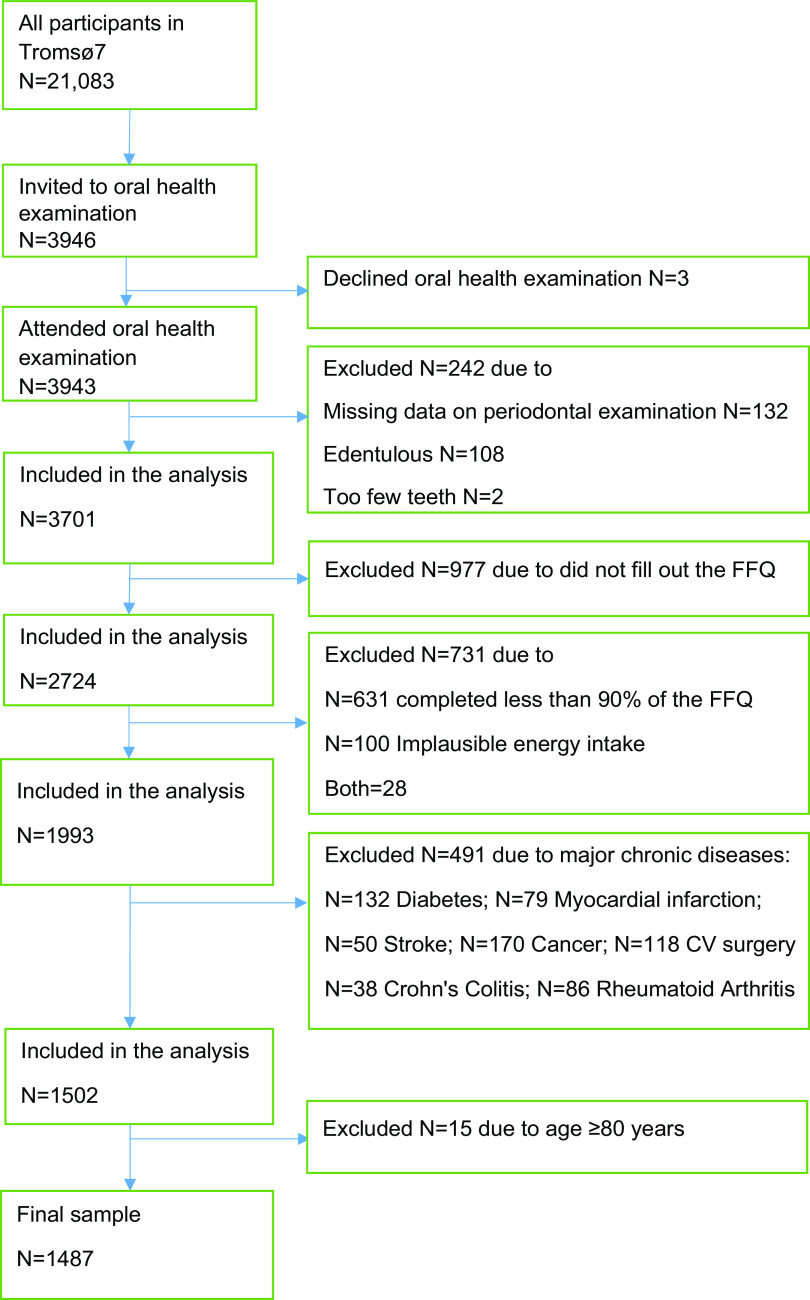
Flow chart of the study sample

As shown in Figure 1, we excluded participants with missing data on periodontal examination and those with too few teeth (< 2) from categorisation as a periodontitis case. We further excluded participants who completed less than 90 % of the FFQ frequency questions^(20)^ and those with implausible daily energy intake (< 500 or > 3500 kcal for women and < 800 or > 4000 kcal for men)^(22)^. Finally, we excluded participants with self-reported diabetes, myocardial infarction, stroke, cancer, coronary surgery/intervention, Crohn’s disease/ulcerous colitis or rheumatoid arthritis (because individuals with these diseases could modify dietary habits) and those aged ≥ 80 years. Thus, the final analytical sample consisted of 1487 participants (55·5 % women) aged 40–79 years.

### Dietary assessment

The FFQ^(21)^ in the Norwegian language was handed out in its paper version to all participants. Participants could choose to complete the FFQ at the examination site or return it by mail. Technical assistance to complete the questionnaires was available at the examination site. The FFQ includes 261 questions on the frequency and amount of intake of various food items, dishes, and beverages, as well as meals and dietary supplements^(19)^. Daily energy intake in kilojoules and food and nutrients in grams (g) were calculated using the nutrient calculation system KBS, with database AE14 at the University of Oslo, based on the Norwegian food composition tables from 2014 to 2015. The calculation of daily nutrient intake included the contribution of food items and dietary supplements.

### Periodontal assessment and case definition of periodontitis

The oral health examination consisted of a clinical and radiographic examination, performed by calibrated dental hygienists. The clinical examination consisted of probing pocket depth, measured to the closest millimetre with a periodontal probe (UNC15 LM1100-EX) at four sites per tooth, including all natural teeth, except third molars and bleeding on probing. An orthopantomogram was used to assess interdental radiographic marginal bone level (RBL). RBL of interproximal surfaces of all teeth, excluding third molars, was measured linearly with a transparent plastic ruler on the orthopantomogram as described by Holde et al.^(23)^ Periodontitis was diagnosed primarily from RBL according to the American Academy of Periodontology and the European Federation of Periodontology classification system of periodontal disease^(24,25)^. To define periodontitis groups, we used an indirect estimation using RBL as a function of age, i.e. per cent radiographic bone loss divided by the age of the participant (% RBL/age) based on the most severely affected interproximal site in the mouth on participants who had interdental bone loss at ≥ 2 non-adjacent teeth. RBL has been demonstrated as the best predictor of future disease in the absence of treatment, reflects disease history at a given age and includes all risk factors that may have affected bone loss over the individual’s lifetime^(26)^. For the present study, to achieve a sufficient number of participants in the subgroups, the thresholds of < 0·25, 0·25–0·75 and > 0·75 were applied. Based on these thresholds, three periodontitis groups were created for analysis: (1) no periodontitis or slow bone loss, (2) moderate bone loss and (3) rapid bone loss, respectively.

### Number of teeth

Number of teeth was categorised as 25–28, 20–24 and ≤ 19. The threshold of ≤19 teeth was chosen in line with the definition of inadequate dentition proposed by the WHO^(27)^. The threshold of twenty-five teeth represents the average number of teeth among participants in the present study sample.

### Covariates

Information on covariates was taken from study questionnaires. Education was categorised as primary (primary/partly secondary: up to 10 years of schooling), secondary (upper secondary: minimum of 3 years) and tertiary (college/university education). Smoking status was categorised as never, former and current smoker. Ever-smoking was estimated by combining former and current smokers. Toothbrushing frequency was categorised as twice/day or more and once/day or less. BMI was calculated as measured weight in kilograms and height in metres squared (kg/m^2^) and categorised as underweight/normal weight (< 25·0 kg/m^2^), overweight (25·0–29·9 kg/m^2^) and obese (≥ 30·0 kg/m^2^). The few participants with underweight (*n* 9) were included in the normal weight group. Physical activity in leisure time was assessed by the Saltin-Grimby Physical Activity Level Scale^(28)^ and categorised as sedentary, light and moderate-to-vigorous. Daily energy intake (kilojoules/d) was divided into tertiles.

### Identification of dietary patterns

**Fig. 2:**
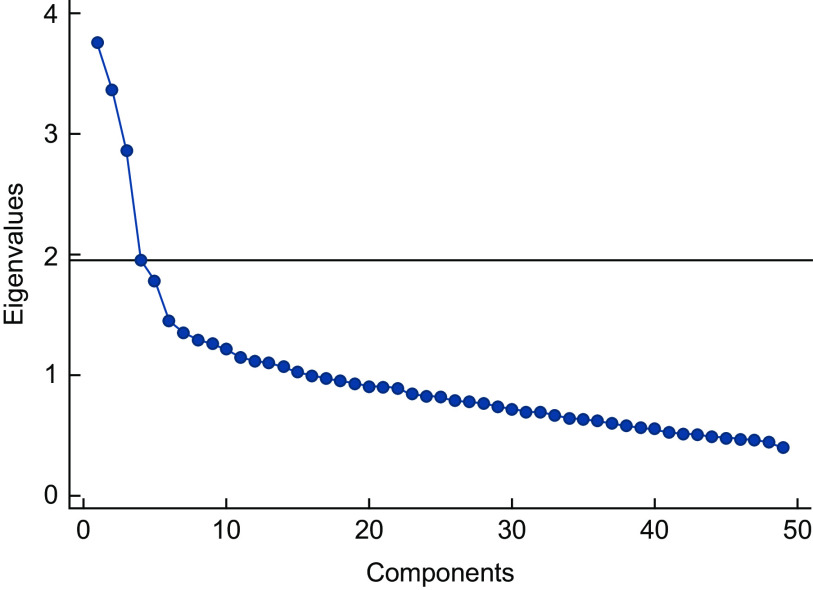
Scree plot for the identification of dietary patterns (components) by principal component analysis. Food intakes (g/d) were aggregated into 49 food groups and used as input variables

Intake of 235 foods and beverages (g/d) was used in the analysis. According to similarities in nutritional composition and usage, single food and beverage intakes were manually aggregated into forty-nine groups (see online supplementary material, Supplemental Table 1). The forty-nine groups were used in a principal component analysis (PCA) with a correlation matrix to identify linear combinations of food groups that explained the greatest variance. Statistical software performs standardisation by default using correlation matrix. The resulting components were rotated orthogonally for interpretability. The number of principal components retained was based on eigenvalues > 1·0, inspection of the scree plot (Fig. 2) and interpretability. Loadings of food category variables > |0·20| were used to characterise principal components as dietary patterns. The Kaiser-Meyer-Olkin measure of sampling adequacy was 0·76, and the Bartlett test of sphericity *P* < 0·001 was satisfactory. When PCA was stratified by sex and age (40–49 *v.* 50–79 years), relatively similar dietary patterns were identified (data not shown). Accordingly, PCA and all subsequent statistical analyses were performed on the entire sample. When we compared participants with valid dietary data, no major chronic diseases, with and without oral examination (*n* 8360) to those in the present study sample (*n* 1487), the same dietary patterns were identified. There were only minor differences in the magnitude of factor loadings and in variability, which was explained by certain principal components. High correlation coefficients between component scores of identical patterns were detected (data not shown). We compared PCA analyses, based on unadjusted and energy-adjusted weights. We chose gram weights as input variables in PCA analysis, as unadjusted patterns were more interpretable. Five energy-adjusted dietary patterns were extracted. Four energy-adjusted patterns had similar loadings and described similar dietary patterns when compared with the unadjusted patterns. However, the fifth energy-adjusted dietary pattern was difficult to interpret. Therefore, we performed energy adjustment later in the analytical process by including energy intake in regression models. A detailed description of energy-adjusted dietary patterns and regression models with them can be found in online supplementary material, Supplemental Appendix 2: Energy-adjusted dietary pattern analysis.

### Statistical analysis

Component dietary pattern scores were split into tertiles (tertile 1 = low intake, tertile 2 = moderate intake, tertile 3 = high intake). We calculated descriptive statistics for the full sample and for those in tertiles 1 and 3 of the identified dietary patterns. We used multinomial logistic regression models to study the association between tertiles of dietary patterns and periodontitis groups and number of teeth adjusted for sex, age, education, smoking status, toothbrushing frequency, BMI, physical activity and energy intake. We presented results as OR with 95 % CI. To test for linear trend across tertiles of dietary patterns, we used the median of each tertile and treated it as a continuous variable. To test the significance of the interaction, we included a product term with the respective stratification variable, i.e. sex, age group (40–49 years *v.* 50–79 years), smoking (never- *v.* ever-smoker), and BMI (< 30·0 kg/m^2^ *v.* ≥ 30·0 kg/m^2^) and the median value of the dietary pattern’s tertiles to test the significance of the interaction. No evidence supporting an interaction effect (*P* < 0·05) was found; thus, these results were not presented.

All analyses were conducted using STATA version 16 (StataCorp, College Station, Texas, USA). All tests were two-tailed, and *P*-values < 0·05 were considered to be statistically significant.

## Results

### Characteristics of the study sample

The study sample comprised 48·6 % participants with moderate bone loss and 6·1 % with rapid bone loss. In total, 8·1 % of participants had 4–19 teeth (Table 1).

**Table 1:** Characteristics of the study population

Characteristics	*n* or Mean	% or s d
Sex		
Women	826	55·5
Age (years)	55·8	9·5
Age group (years)		
40–49	456	30·6
Education level^*^		
Primary	252	17·1
Secondary	420	28·5
Tertiary	801	54·4
Smoking status		
Never	681	46·4
Former	595	40·5
Current	192	13·1
Toothbrushing frequency		
Once/day or less often	260	17·6
BMI category (kg/m^2^)		
Underweight (< 18·5)	9	0·6
Normal weight (18·5–24·9)	510	34·4
Overweight (25·0–29·9)	669	45·1
Obesity (≥ 30)	296	19·9
Physical activity		
Sedentary	187	12·8
Light	820	56·2
Moderate-to-vigorous	452	31·0
Energy intake (kilojoules/d)	9338	2636
Periodontitis group		
No periodontitis	176	11·8
Slow bone loss	497	33·5
Moderate bone loss	723	48·6
Rapid bone loss	91	6·1
Number of teeth	25·1	4·2
Number of teeth categories		
25–28 teeth	1·047	70·4
20–24 teeth	319	21·5
4–19 teeth	121	8·1

Values are numbers (percentages) for categorical variables and mean (sd) for continuous variables.

* Low (primary/partly secondary: up to 10 years of schooling), medium (upper secondary: minimum of 3 years) and high (college/university education).

### Characteristics of dietary patterns

Figure 2 shows the scree plot for identification of dietary patterns. Four dietary patterns that explained 24·3 % of the total variability in food intake were identified and labelled ‘Fruit and vegetables’, ‘Westernised’, ‘Meat/fish and potatoes’ and ‘Refined grain and dessert’ (Table 2) based on the highest food group loadings. For the fruit and vegetables pattern, the following food groups loaded ≥|0·20|: vegetables, fruit, berries, dried fruit, nuts or peanut butter, fatty fish, seafood, vegetarian dish, beans/lentils and stew/soup with fish. The Westernised pattern was characterised by high intake of processed meat dishes or fast food, rice or pasta, salty snacks, chicken, fried potato dishes, tomato sauces, salad dressing and wok with meat/chicken. The meat/fish and potatoes pattern loaded positively for red meat, sausage or bacon, stew with meat or chicken, lean fish, processed fish, boiled/baked or mashed potatoes, and sauce butter/margarine melted or creamy dressing. The refined grain and dessert pattern loaded positively for food high in refined grains, sweet spreads or sweeteners, food containing 50–100 % whole grains, cakes or dessert, butter, margarine or mix of butter, margarine and oil as a spread, whey cheese and negatively for wine.

**Table 2:** Loading matrix (≥ |0·20|) and explained variances for the first four PCs identified by PCA

	Fruit and vegetables	Westernised	Meat/fish and potatoes	Refined grain and dessert
Food group	PC1	PC2	PC3	PC4
Refined grains				0·30
Whole grains 50–100 %				0·34
Butter, margarine, or mix of butter, margarine, and oil as a spread				0·24
Whey cheese				0·26
White cheese				
Processed meat or pate´ for sandwiches				
Canned/smoked fish, cod roe from a toothpaste-like tube or shrimp/crab for sandwiches				
Sweet spreads or sweeteners				0·36
Mayonnaise salads or mayonnaise as spread/dressing				
Regular milk/soured milk or low-fat milk				0·22
Natural/flavoured yoghurt or milk with probiotics				
Flavoured chocolate/strawberry milk, milk/cream in coffee/tea or hot chocolate/cocoa				
Water				
Juice				
Sugary drinks (ice tea, soft drinks or fruit/berry drinks)				
Artificially sweetened drinks (ice tea, soft drinks, or fruit/berry drinks) and non-alcoholic beer				
Wine				−0·23
Alcoholic beverages except wine				
Tea				
Coffee				
Sausage or bacon			0·33	
Processed meat dishes or fast food		0·36		
Red meat			0·36	
Stew meat and chicken			0·29	
Chicken		0·28		
Processed fish			0·27	
Lean fish			0·33	
Fatty fish	0·22			
Shrimp/crab or seafood wok	0·22			
Vegetarian dish or soup vegetable	0·22			
Eggs and egg dishes				
Boiled/baked or mashed potatoes			0·33	
Fried potatoes		0·20		
Rice or pasta		0·35		
Vegetables	0·34			
Root vegetables (onion, carrot, rutabaga)	0·26			
Fruit	0·33			
Berries	0·27			
Dried fruit or fruit and nut mix	0·24			
Dessert or cakes				0·26
Chocolate or candy				
Salty snacks		0·27		
Nuts or peanut butter	0·23			
Sauces, sauce butter/margarine melted, or creamy dressing			0·26	
Salad dressing like Thousand Island, salad dressing oil, mustard, or soy sauce		0·24		
Tomato sauce		0·33		
Wok with meat/chicken		0·24		
Beans/lentils	0·21			
Stew/soup with fish	0·21			
Proportion of variance explained by each dietary pattern, %	6·8	6·7	6·1	4·7
Cumulative	24·3			

PC: Principal Components; PCA: Principal Component Analysis.

Characteristics of the study population according to tertiles of dietary pattern are shown in Table 3. Participants in tertile 3 of the fruit and vegetables pattern were more likely to be women, have tertiary education, be never-smokers and be more physically active. Participants in tertile 3 of the Westernised pattern were more likely to be men, younger and have tertiary education. Participants in tertile 3 of the meat/fish and potatoes pattern were more likely to be men, older, have primary or secondary education and be ever-smokers. Participants in tertile 3 of the refined grain and dessert pattern were more likely to be men and never-smokers.

**Table 3:** Characteristics of the study population according to tertiles of dietary pattern scores

	Fruit and vegetables						Westernised						Meat/fish and potatoes						Refined grain and dessert					
Characteristics	Tertile 1			Tertile 3			Tertile 1			Tertile 3			Tertile 1			Tertile 3			Tertile 1			Tertile 3		
	%	Mean	sd	%	Mean	sd	%	Mean	sd	%	Mean	sd	%	Mean	sd	%	Mean	sd	%	Mean	sd	%	Mean	sd
Sex																								
Women	42·5			65·7			64·7			45·3			70·8			38·6			69·0			42·2		
Men	57·5			34·3			35·3			54·7			29·2			61·4			31·0			57·8		
Age group (years)																								
40–49	36·7			24·9			7·1			57·6			42·9			20·8			32·7			30·5		
50–79	63·3			75·1			92·9			42·4			57·1			79·2			67·3			69·5		
Education level^*^																								
Primary	22·5			11·5			27·3			8·6			11·2			23·1			16·6			20·3		
Secondary	30·0			26·9			30·8			26·7			20·0			37·2			27·0			31·3		
Tertiary	47·5			61·6			42·0			64·8			68·8			39·7			56·4			48·4		
Smoking status																								
Never	38·9			50·5			40·6			52·5			54·3			37·5			38·7			51·5		
Former	40·0			42·6			44·9			35·0			36·7			45·2			46·0			36·7		
Current	21·1			6·9			14·5			12·4			9·0			17·3			15·3			11·8		
Toothbrushing frequency																								
Twice/day or more often	77·0			88·6			81·0			83·3			88·4			77·1			84·6			79·0		
Once/day or less often	23·0			11·4			19·0			16·7			11·6			22·9			15·4			20·9		
BMI category (kg/m^2^)																								
< 25·0	31·0			38·7			39·0			30·6			44·4			28·1			35·6			33·9		
25·0–29·9	48·0			43·1			42·4			47·6			42·0			44·5			44·9			47·3		
≥ 30·0	21·1			18·2			18·6			21·9			13·5			27·3			19·4			18·8		
Physical activity																								
Sedentary	21·2			7·6			12·0			15·1			10·5			15·1			12·9			13·6		
Light	53·5			56·3			65·5			46·7			54·3			56·3			58·7			55·1		
Moderate-to-vigorous	25·3			36·1			22·5			38·2			35·2			28·6			28·4			31·3		
Energy intake (kilojoules/d)		8634	2489		10 279	2593		7705	2035		11 043	2477		8110	2367		10 768	2411		7928	2395		11 067	2315
Periodontitis group																								
No periodontitis	10·9			11·3			6·5			17·8			14·7			8·5			12·9			12·3		
Slow bone loss	29·6			36·4			27·6			40·6			37·5			30·1			32·7			31·5		
Moderate bone loss	50·6			48·9			59·5			36·8			42·3			53·1			47·1			50·3		
Rapid bone loss	8·9			3·4			6·4			4·8			5·4			8·3			7·3			5·9		
Number of teeth categories																								
25–28 teeth	66·5			70·3			54·0			83·2			77·4			64·2			75·4			65·3		
20–24 teeth	22·8			19·2			28·6			17·5			15·7			23·4			19·6			23·8		
≤ 19 teeth	10·7			6·5			17·3			1·0			5·0			12·3			5·0			10·9		

Values are percentages for categorical variables and mean (sd) for continuous variables.

* Low (primary/partly secondary: up to 10 years of schooling), medium (upper secondary: minimum of 3 years) and high (college/university education).

### Association between dietary patterns and periodontitis

Compared to tertile 1 of the fruit and vegetables pattern, those in tertile 3 had lower odds of periodontitis characterised by rapid bone loss (OR 0·49, 95 % CI 0·25, 0·98, *P* = 0·043; *P* trend = 0·050) after adjustment for confounders (Table 4).

**Table 4:** Regression models between tertiles of dietary pattern scores and periodontitis groups

Dietary pattern	Moderate bone loss *v.* no periodontitis/slow bone loss				Rapid bone loss *v.* no periodontitis/slow bone loss				Rapid bone loss *v.* moderate bone loss			
	OR	95 % CI	*P*-value	*P* trend^*^	OR	95 % CI	*P*-value	*P* trend^*^	OR	95 % CI	*P*-value	*P* trend^*^
Fruit and vegetables												
Tertile 1 (ref.)	1				1							
Tertile 2	0·76	0·57, 1·02	0·072		0·80	0·58, 1·10	0·164		0·98	0·57, 1·69	0·952	
Tertile 3	0·75	0·43, 1·30	0·307	0·228	0·49	0·25, 0·98	0·043	0·050	0·62	0·31, 1·21	0·161	0·156
Westernised												
Tertile 1 (ref.)	1				1							
Tertile 2	1·02	0·74, 1·39	0·909		0·79	0·54, 1·15	0·220		0·96	0·53, 1·74	0·898	
Tertile 3	0·98	0·53, 1·81	0·947	0·168	0·53	0·24, 1·16	0·110	0·085	0·67	0·31, 1·46	0·309	0·287
Meat/fish and potatoes												
Tertile 1 (ref.)	1				1							
Tertile 2	0·93	0·69, 1·25	0·627		0·88	0·63, 1·25	0·480		0·69	0·36, 1·31	0·255	
Tertile 3	0·64	0·34, 1·22	0·178	0·470	1·06	0·55, 2·07	0·858	0·697	1·20	0·62, 2·32	0·580	0·445
Refined grain and dessert												
Tertile 1 (ref.)	1				1							
Tertile 2	0·95	0·71, 1·26	0·704		1·00	0·72, 1·39	0·980		0·85	0·48, 1·51	0·575	
Tertile 3	0·80	0·45, 1·44	0·459	0·988	0·93	0·49, 1·79	0·836	0·808	0·93	0·49, 1·77	0·823	0·799

All models adjusted for sex, age, education, smoking status, toothbrushing frequency, BMI, physical activity and energy intake.

* To test for linear trend across tertiles of the dietary pattern score, we used the median of each tertile and treated the variable as continuous variable.

### Association between dietary patterns and number of teeth

Participants with ≤ 19 teeth were more likely to have a diet characterised by higher intake of foods included in the refined grain and dessert pattern (Table 5). Participants in tertile 2 had 2·10-fold increased odds of having ≤ 19 teeth than 25–28 teeth. Participants in tertile 3 of the refined grain and dessert pattern had 2·38- and 3·52-fold increased odds of having ≤ 19 teeth than 20–24 and 25–28 teeth, respectively (Table 5). Foods correlated with the Westernised pattern were consumed less often by participants with ≤ 24 teeth (Table 5).

**Table 5:** Regression models between tertiles of dietary pattern scores and number of teeth categories

Dietary pattern	OR	95 % CI	*P*-value	*P* trend^*^	OR	95 % CI	*P*-value	*P* trend^*^	OR	95 % CI	*P*-value	*P* trend^*^
Fruit and vegetables	≤ 19 teeth *v.* 25–28 teeth				≤ 19 teeth *v.* 20–24 teeth				20–24 teeth *v.* 25–28 teeth			
Tertile 1 (ref.)	1				1				1			
Tertile 2	0·81	0·45, 1·43	0·464		0·89	0·49, 1·61	0·703		0·90	0·64, 1·28	0·568	
Tertile 3	0·84	0·44, 1·62	0·605	0·620	1·06	0·54, 2·08	0·867	0·860	0·79	0·54, 1·16	0·233	0·234
Westernised^†^	≤ 24 teeth v. 25–28 teeth											
Tertile 1 (ref.)	1											
Tertile 2	0·62	0·45, 0·86	0·004									
Tertile 3	0·58	0·38, 0·89	0·013	0·208								
Meat/fish and potatoes	≤ 19 teeth *v.* 25–28 teeth				≤ 19 teeth *v.* 20–24 teeth				20–24 teeth *v.* 25–28 teeth			
Tertile 1 (ref.)	1				1				1			
Tertile 2	0·64	0·32, 1·25	0·189		0·61	0·30, 1·21	0·156		1·05	0·74, 1·49	0·792	
Tertile 3	0·80	0·40, 1·62	0·537	0·760	1·01	0·49, 2·10	0·971	0·707	0·79	0·52, 1·19	0·260	0·226
Refined grain and dessert	≤ 19 teeth *v.* 25–28 teeth				≤ 19 teeth *v.* 20–24 teeth				20–24 teeth *v.* 25–28 teeth			
Tertile 1 (ref.)	1				1				1			
Tertile 2	2·10	1·09, 4·06	0·027		1·73	0·88, 3·40	0·110		1·21	0·86, 1·72	0·278	
Tertile 3	3·52	1·69, 7·33	0·001	0·001	2·38	1·12, 5·07	0·024	0·026	1·48	1·00, 2·19	0·053	0·053

All models adjusted for sex, age, education, smoking status, toothbrushing frequency, BMI, physical activity and energy intake.

* To test for linear trend across tertiles of the dietary pattern score, we used the median of each tertile and treated the variable as continuous variable.

† For model with tertiles of Westernised dietary pattern score 20–24 teeth and ≤ 19 teeth categories were combined, and a dichotomous variable was used (≤ 24 teeth *v.* 25–28 teeth) as there were few participants (*n* 5 (1·0 %)) who had ≤ 19 teeth and were categorised into third tertile of the Westernised dietary pattern.

## Discussion

We aimed to explore the association between dietary patterns and (1) periodontitis and (2) number of teeth, in a population-based sample from Norway. Out of four identified dietary patterns, the fruit and vegetables pattern was associated with 50 % lower odds of the periodontitis characterised by rapid bone loss. The refined grain and dessert pattern was associated with having ≤ 19 teeth.

Diet is a complex exposure; combinations of food groups or nutrients may have different effects when compared to single food and nutrient exposures. A study by Blostein et al.^(29)^ demonstrated that, when food groups that had the highest loadings for dietary patterns associated with caries were tested as predictors in diet-caries associations, no significant relations were found; however, dietary patterns were found to be associated with caries. Our findings are consistent with the study by Wright et al.^(8)^, which used a data-driven approach to derive dietary patterns using food groups. Their study found associations between a dietary pattern rich in salad, fruit, vegetables, poultry, seafood, water and tea and a lower extent of clinical attachment loss. In the present study, the fruit and vegetables pattern was associated with periodontitis and included similar food groups. A more recent cross-sectional study reported that the ‘high micronutrient (i.e. *β*-carotene, vitamin B_6_, folate, vitamin C, vitamin E, iron, potassium and magnesium) and fibre’ nutrient pattern assessed by *the posteriori* approach using nutrient intakes was associated with reduced risk of self-reported periodontal disease^(30)^. We can expect that the intake of these nutrients is higher for those in tertile 3 of the fruit and vegetables pattern. Moreover, fruits and vegetables contain high levels of phytochemicals, bioactive components that may contribute to the beneficial effects of healthy diets. The Western or Prudent dietary patterns are often derived from exploratory patterns, and these patterns were also identified in the present study^(31)^. However, exploratory patterns identified in different groups and populations can vary and have different levels of reproducibility, making comparison of studies difficult. Nevertheless, our findings were in line with previous studies and complemented those of hypothesis-driven methods.

It has been suggested that diet may affect periodontitis by shaping the microbiota and modulating systemic low-grade inflammation^(4,32)^. Further, nutrients are involved in the formation of bones and teeth, and nutrients act as antioxidants, methyl donors and cofactors that can affect DNA methylation and contribute to the reduction of DNA damage^(33)^. Many vitamins and trace elements, such as vitamins A, D, C, E, B_6_, and B_12_, folate, zinc, iron, copper and selenium, play an important role in the immune response to infection^(34)^. In addition to micronutrient deficiencies^(35)^, macronutrient imbalance, for example an increased consumption of refined carbohydrates and low fibre intake, may be involved in the pathogenesis of periodontitis^(36,37)^.

Recent studies have indicated that high intake of fermentable carbohydrates (mainly sucrose) not only significantly increases the risk of caries but is also associated with the risk of periodontal disease^(38)^. No association could be confirmed between the refined grain and dessert pattern and periodontitis in the present study. However, the refined grain and dessert dietary pattern was associated with inadequate dentition. Dental caries and periodontitis are the major causes of tooth loss, but the mechanisms by which excessive intake of carbohydrates relates to caries and periodontitis are supposed to be different. The development of caries requires sugars and acidogenic, acid-tolerant bacteria^(39)^. Demineralisation of the enamel occurs when plaque bacteria metabolise dietary sugars and produce organic acids, which increase the solubility of the calcium hydroxyapatite that is present in the hard tissue of teeth. When it comes to periodontitis, sugars may confer pro-inflammatory properties to microbiota in the mouth and gut, thus contributing to local and systemic inflammation^(4,40)^. It has been suggested that the dysbiosis of the oral microbiota may trigger changes in the gut microbiota, which creates a higher predisposition for the development of various chronic diseases. We have previously shown that periodontitis was associated with cardiovascular risk and higher C-reactive protein concentrations^(41)^. Owing to the relation between diet and chronic inflammation, several studies reported associations between Dietary Inflammatory Index and periodontitis^(6,7,11)^ and more lost teeth^(17)^.

The present study is cross-sectional; therefore, we cannot draw conclusions about causal relationships between diet and oral health outcomes. Reverse causation can occur when people change their diet due to inadequate dentition or oral health disease. In the present study, 8·1 % of individuals had ≤ 19 teeth. Previous studies have shown that severe tooth loss and masticatory impairment may result in dietary changes like limited consumption of fruits and vegetables, and increased consumption of sugary and easy-to-chew foods^(42,43)^. We tried to account for this challenge by performing regression analysis on the associations between the fruit and vegetables pattern and periodontitis after the exclusion of individuals with ≤ 19 teeth, and similar results were observed (data not shown). Moreover, some adults with periodontitis might change their dietary habits due to pain, discomfort, high dentinal hypersensitivity^(44)^, impaired senses of smell and taste,^(45)^ or other reasons. One study reported that the periodontitis group avoided alcohol, sweets, carbonated beverages, hot and cold drinks, cold food, and hard textured and fibrous foods more often than controls^(46)^. We also found that the Westernised dietary pattern was less common among participants with ≤ 24 teeth. We observed that the group with ≤ 24 teeth included a higher percentage of older than younger participants (40–49 years: *n* 67, 14·7 %; 50–59 years: *n* 95, 19·4 %; 60–69 years: *n* 188, 46 %; 70–79 years: *n* 440, 67·7 %; data not shown).

Previous studies found a modifying effect of obesity and smoking on the association between poor diet and periodontitis^(5,10,47)^. We found no evidence of an interaction effect of obesity and smoking on the association between dietary patterns and periodontitis (data not shown).

Definition of periodontitis was based on the 2017 World Workshop on the Classification of Periodontal and Peri-Implant Diseases and Conditions, which is the most recent classification. Tonetti & Claffey^(48)^ suggested that the periodontitis progression case definition that demonstrates longitudinal attachment loss should be considered in risk factor research. By using the rate of bone loss related to age rather than stages, we were able to consider disease susceptibility due to life-long exposure to different causal factors, including established, modifiable risk factors like smoking and dysglycaemia,^(26)^, and to achieve smaller age differences between the periodontitis groups. We used grading to identify periodontitis cases. Due to the few participants with grade C, and to get a more statistically robust group with advanced periodontitis, we slightly modified the cut-off and labelled the different levels of bone loss as slow, moderate, and rapid^(26)^.

### Strengths

The present study has several strengths. The Tromsø Study is a well-designed population-based cohort where data collection was performed by trained personnel using standardised protocols and instruments. To our knowledge, this is the first study that relates *a posteriori* food group-based dietary patterns and two objectively measured oral health outcomes (periodontitis and number of teeth). Assessments of dietary intake were based on a previously validated FFQ that captures the habitual dietary intake, and strict criteria were applied to exclude participants with unreliable dietary data. In addition, participants with major chronic diseases, including diabetes, (which might lead to changes in dietary habits) were excluded from the analysis. These exclusions were made to improve the internal validity of the present study; however, exclusions may also have resulted in less generalisable results. One study demonstrated the effect of interactions between diabetes and dietary patterns on periodontitis^(49)^. Participants who consumed an anti-inflammatory diet and did not have diabetes experienced the lowest risks of periodontitis and tooth loss. However, in the context of diabetes, the efficacy of such a diet may be weakened or even eliminated. The main results based on unadjusted patterns were comparable with energy-adjusted patterns (see online supplementary material, Supplemental Appendix).

### Limitations

The main limitation is the cross-sectional design of the present study, which left us unable to determine the direction of the relationships between dietary patterns and outcomes. Bias due to self-reporting and selection bias may also have occurred. The present study is observational in nature; therefore, the observed associations might be explained by unmeasured or residual confounding. PCA is the most commonly used data-driven reduction technique to identify dietary patterns. However, the limitations of PCA include subjectivity related to the selection of food groups and the determination of retained components, limited reproducibility in different populations, correlations/interactions of components with many lifestyle characteristics, and that the retained patterns can only explain part of the total variation in food intake, i.e. 24 % in our study^(15)^. There are some indications of an interaction between genetic risks for age-related diseases and dietary patterns^(50)^; however, it is not known if this is the case for oral diseases.

## Conclusion

Our study contributes to the evidence that overall diet may be associated with advanced periodontitis and tooth loss. Oral hygiene routines, periodontal treatment, and smoking cessation are recommended for patients with periodontitis, but dietary recommendations have not yet been developed due to limited evidence on the causal relationship. More likely, associations between dietary patterns and periodontitis are bidirectional and can be impacted by additional risk factors. Our findings are in line with current official chronic disease prevention dietary guidelines that encourage eating less foods rich in refined grains, sugar and saturated fats – especially processed foods – and eating more vegetables, fruits, berries, nuts, fish and legumes. To what degree diet is an essential component in the prevention of chronic inflammatory diseases remains uncertain; further prospective studies that measure the effectiveness of dietary interventions on periodontitis are necessary.

## Supporting information

Petrenya et al. supplementary material Petrenya et al. supplementary material

## Supplementary material

For supplementary material accompanying this paper visit https://doi.org/10.1017/S1368980023002690
