# Supplementary material for: Empirically derived dietary patterns in relation to periodontitis and number of teeth among Norwegian adults
Source: Public Health Nutr. 2024 Jan 15;27(1):e27. doi: 10.1017/S1368980023002690 (PMC10830357; doi:10.1017/S1368980023002690)
Supplement: Petrenya et al. supplementary material [file S1368980023002690sup001.docx]

**Supplementary Appendix**

1. **Characteristics of food groups**

**Supplementary Table 1** Characteristics of food groups

| Food groups | Food items, g/day |
| --- | --- |
|  |  |
|  |  |
| 1. Refined grains | Bread, white  Crispbread, white  Sweet bun/roll/pretzel  Waffle  Pancakes  Noodles, instant (e.g. Mr Lee)  Cereal rice/oats, puffed  Cereal muesli, sweetened  Cornflakes  Cereal, sweetened (e.g. Honnikorn/Choco/Frosties)  Porridge with rice/other with milk  Porridge sour cream  Hotdog bun/potato wrap |
| 2. Whole grains 50-100% | Bread, wholegrain 50%  Bread, wholegrain 50-100%  Crispbread, wholegrain  Porridge oatmeal  Oatmeal/4-grain  Cereal muesli, unsweetened  Cereal, unsweetened (e.g. All Bran/Weetabix/Havrefras) |
| 3. Butter, margarine, or mix of butter, margarine, and oil as spread | Butter on bread  Margarine Bremykt on bread  Margarine Brelett on bread  Margarine soft other on bread (e.g. Soft Flora/Soft Ekstra)  Margarin Vita Hjertego on bread  Margarine Soft Flora Lett/Vita Hjertego Lett on bread  Margarine Melange on bread  Margarine other on bread |
| 4. Whey cheese | Spread cheese, whey/prim  Spread cheese, whey/prim light |
| 5. White cheese | Spread cheese, white/gouda  Spread cheese, white/gouda light  Spread cheese, soft spread  Spread cheese, soft spread light  Spread cheese, cottage  Spread cheese, blue |
| 6. Processed meat or paté for sandwiches | Spread, bologna sausage  Spread, boiled ham/turkey ham/light saveloy  Spread, salami/smoked/cured sausage  Spread, liver paste  Spread, liver paste light |
| 7. Canned/smoked fish, cod roe from a toothpaste-like tube, or shrimp/crab for sandwiches | Spread, caviar  Spread, caviar and cod liver mix  Spread, mackerel in tomato sauce  Spread, smoked/gravlax salmon/trout  Spread, sardines/pickled herring/anchovy  Spread, tuna fish  Spread, shrimp/crab |
| 8. Sweet spreads or sweeteners | Spread, jam/marmalade  Spread, jam light  Spread, chocolate/nut  Spread sweet (e.g. honey/syrup/Sunda)  Jam on cereal/porridge  Sugar on cereal/porridge  Sugar in coffee  Sugar in tea |
| 9. Mayonnaise salads or mayonnaise as spread/dressing | Mayonnaise on bread  Spread, mayonnaise salad (e.g. Italian salad)  Spread, mayonnaise salad light (e.g. Italian salad light)  Potato salad with mayonnaise  Mayonnaise/remoulade  Mayonnaise/remoulade light |
| 10. Regular milk/soured milk or low-fat milk | Milk, whole milk/cultured/kefir 3.5% fat  Milk, semi-skimmed 1% fat  Milk, extra semi-skimmed 0.5% fat fortified with vitamin D  Milk, skimmed/cultured skimmed 0.1% fat |
| 11. Natural/flavoured yoghurt or milk with probiotics | Yoghurt, natural  Yoghurt, drinkable  Yoghurt, fruit  Yoghurt, GoMorgen musli  Yoghurt, light musli  Yoghurt, light fruit  Milk with probiotics Biola/Cultura flavoured  Milk with probiotics Biola/Cultura natural |
| 12. Flavoured chocolate/strawberry milk, milk/cream in coffee/tea or hot chocolate/cocoa | Milk, flavoured chocolate/strawberry  Milk/cream in coffee/tea  Hot chocolate/cocoa |
| 13. Water | Water, tap  Water, bottle still/sparkling |
| 14. Juice | Juice, orange  Juice, apple/other  Nectar, apple/other |
| 15. Sugary drinks (ice tea, soft drinks or fruit/berry drinks) | Ice tea, sugar  Soft drinks, sugar  Fruit/berry drinks, sugar |
| 16. Artificially sweetened drinks (ice tea, soft drinks or fruit/berry drinks) and beer non-alcoholic | Ice tea, artificially sweetened  Soft drinks, artificially sweetened  Fruit/berry drinks, artificially sweetened  Beer, non-alcoholic ≤0.7% alcohol |
| 17. Wine | Wine, red  Wine, white |
| 18. Alcoholic beverages except wine | Beer, strong >4.7% alcohol/pils 4.5% alcohol  Beer, light ≤2.7% alcohol  Cider/alcopops with alcohol  Wine, fortified (e.g. sherry/port)  Liquor/spirits  Drinks, mixed/cocktail |
| 19. Tea | Tea, black  Tea, green  Tea, herbal |
| 20. Coffee | Coffee, boiled/French press  Coffee, filtered  Coffee, instant  Coffee espresso  Coffee, cafe latte  Coffee, cappuccino |
| 21. Sausage or bacon | Sausage, pork/beef  Sausage, pork/beef light  Sausage, chicken/turkey  Bacon |
| 22. Processed meat dishes or fast food | Sausage hot dog, pork/beef  Sausage hot dog, chicken/turkey  Hamburger with bun  Taco, shell with meat and salad  Tortilla wrap with meat and salad  Kebab  Lasagne/moussaka  Calzone  Pie/quiche  Spring rolls  Pizza  Meat burger karbonade  Meat burger/medisterburger/meatloaf  Stew with minced meat/meat sauce |
| 23. Red meat | Roast moose/deer/reindeer/venison  Chops pork/beef/lamb  Roast pork/beef/lamb  Roast moose/deer/reindeer/venison |
| 24. Stew meat and chicken | Stew with meat/frikasse/faarikaal  Stew with meat and vegetables/betasuppe  Chicken stew |
| 25. Chicken | Chicken, grilled  Chicken, fillet  Chicken, stew |
| 26. Processed fish | Fish burger/pudding  Fish balls  Fish sticks  Fish baked gratin |
| 27. Lean fish | Cod/pollock/haddock/catfish/redfish, boiled  Cod/pollock/haddock/catfish/redfish, fried |
| 28. Fatty fish | Herring, fresh/smoked/cured  Mackerel, fresh/smoked  Salmon/trout, boiled/fried |
| 29. Shrimp/crab or seafood wok | Shrimp/crab  Wok with seafood and vegetables |
| 30. Vegetarian dish or vegetable soup | Vegetarian dish/pizza/gratin  Soup, vegetable (e.g. tomato/cauliflower/pea) |
| 31. Eggs and eggs dishes | Omelette  Egg, boiled/fried/scrambled |
| 32. Boiled/baked or mashed potatoes | Potatoes, boiled/baked  Potatoes, mashed |
| 33. Fried potatoes | Potatoes, gratinated with cream  Potatoes, fried  French fries, deep-fried  French fries, oven-baked |
| 34. Rice or pasta | Rice  Pasta, spaghetti/macaroni/other |
| 35. Vegetables | Cabbage  Cauliflower  Broccoli  Brussel sprouts  Lettuce (e.g. iceberg/arugula)  Bell/sweet pepper  Avocado  Tomato  Corn  Spread, vegetables  Salad, mixed (e.g. with lettuce, tomato, cucumber, corn)  Vegetables, mixed frozen |
| 36. Root vegetables (onion, carrot, rutabaga) | Onion, raw/fried  Carrot  Rutabaga |
| 37. Fruit | Apple  Pear  Banana  Orange  Clementine  Grapefruit  Peach/nectarine  Kiwi  Grape  Melon  Fruit on bread  Fruit salad, fresh |
| 38. Berries | Strawberry, fresh/frozen  Raspberry, fresh/frozen  Blueberry  Cloudberry |
| 39. Dried fruit or fruit and nut mix | Raisin  Fruit, dried (e.g. apricot/fig)  Fruit and nut mix |
| 40. Dessert or cakes | Pudding (e.g. chocolate/caramel)  Sauce, vanilla  Cream, whipped  Ice cream  Ice lolly/sorbet  Fruit, hermetic  Sweet bun/roll with custard  Danish pastry  Muffin/cake without icing  Lefse with spread  Cake, chocolate/brownie  Cake, sponge with whipped cream/marzipan  Cake, chocolate/brownie  Biscuit, sweet (e.g. Cookies, Bixit, Hop Nobs)  Snowball (chocolate-coated marshmallow treat with dried coconut) |
| 41. Chocolate or candy | Chocolate (e.g. milk chocolate/Snickers)  Chocolate, dark ≥70% cocoa  Confections, chocolate  Candy/pastilles/liquorice/Seigmenn  Candy/sweets mix smagodt  Pastilles, sugar-free |
| 42. Salty snacks | Potato chips  Salty snacks, other |
| 43. Nuts or peanut butter | Peanut butter  Almond/hazelnut/walnut  Peanut/cashew |
| 44. Sauces, sauce butter/margarine melted, or creamy dressing | Sauce, brown/bechamel  Sauce, bearnaise/hollandaise  Butter with herbs/spices  Sour cream 35% fat  Sour cream light 20% fat  Sour cream extra light 10% fat  Sauce butter/margarine melted |
| 45. Salad dressing like thousand Island, salad dressing oil, mustard or soy sauce | Salad dressing (e.g. Thousand Island)  Salad dressing, light (e.g. Thousand Island light)  Salad dressing, oil/vinaigrette  Pesto  Oil olive/other on bread  Mustard  Soy sauce |
| 46. Tomato sauce | Salsa/tomato sauce  Ketchup |
| 47. Wok with meat/chicken | Wok with meat/chicken and vegetables |
| 48. Beans/lentils | Beans/lentils |
| 49. Stew/soup with fish | Stew/soup with fish |

1. **Energy-adjusted dietary pattern analyses**

We used the residual method for energy adjustment ^(1, 2)^, where we generated standardised residuals by regressing the consumption of food groups (g/day) on total energy intake. Standardised residuals were input variables in principal component analysis (PCA) with varimax rotation. All food groups were log-transformed in order to reduce skewness. To account for zeroes in dietary variables, a log (x + 0.1) transformation was applied.

**2.1 Five retained energy-adjusted dietary patterns**

First, five energy-adjusted dietary patterns were extracted based on eigenvalues >1.0 and inspection of the scree plot (Supplementary Figure 1).

**Supplementary Figure 1** Scree plot for identification of five energy-adjusted dietary patterns (components) by principal component analysis (Bartlett test of sphericity, p<0.001; Kaiser-Meyer-Olkin Measure of Sampling Adequacy=0.775).

Supplementary Table 2 shows the loading matrix (≥|0.20|) and explained variances for the first five energy-adjusted dietary patterns.

**Supplementary Table 2** Loading matrix (≥|0.20|) and explained variances for the first five energy-adjusted dietary patterns

|  | Westernised | Fruit and vegetables | Meat/fish  and potatoes | Seafood, eggs, and wine | Refined grain and dessert |
| --- | --- | --- | --- | --- | --- |
|  | PC1 | PC2 | PC3 | PC4 | PC5 |
| Refined grains |  |  |  |  | 0.39 |
| Whole grains 50-100% |  |  |  | -0.22 |  |
| Butter, margarine, or mix of butter, margarine, and oil as spread |  |  |  |  |  |
| Whey cheese |  |  |  |  |  |
| White cheese |  |  |  |  |  |
| Processed meat or paté for sandwiches |  |  |  |  |  |
| Canned/smoked fish, cod roe from a toothpaste-like tube or shrimp/crab for sandwiches |  |  |  | 0.28 |  |
| Sweet spreads or sweeteners |  |  |  |  | 0.34 |
| Mayonnaise salads or mayonnaise as spread/dressing |  |  |  | 0.20 |  |
| Regular milk/soured milk or low-fat milk |  |  |  |  |  |
| Natural/flavoured yoghurt or milk with probiotics |  |  |  |  |  |
| Flavoured chocolate/strawberry milk, milk/cream in coffee/tea or hot chocolate/cocoa |  |  |  |  | 0.22 |
| Water |  |  |  |  |  |
| Juice |  |  |  |  | 0.23 |
| Sugary drinks (ice tea, soft drinks or fruit/berry drinks) |  | -0.26 |  |  |  |
| Artificially sweetened drinks (ice tea, soft drinks or fruit/berry drinks) and non-alcoholic beer | 0.25 |  |  |  |  |
| Wine |  |  |  | 0.30 |  |
| Alcoholic beverages except wine |  | -0.20 |  |  |  |
| Tea |  |  |  |  |  |
| Coffee |  | 0.20 |  |  |  |
| Sausage or bacon |  |  | 0.33 |  |  |
| Processed meat dishes or fast food | 0.32 |  | 0.26 |  |  |
| Red meat |  |  | 0.31 |  |  |
| Stew meat and chicken |  |  | 0.25 |  |  |
| Chicken | 0.31 |  |  |  |  |
| Processed fish |  |  | 0.28 |  |  |
| Lean fish |  |  | 0.26 |  |  |
| Fatty fish |  |  |  | 0.21 |  |
| Shrimp/crab or seafood wok |  |  |  | 0.38 |  |
| Vegetarian dish or soup vegetable |  |  |  |  |  |
| Eggs and eggs dishes |  |  |  | 0.27 |  |
| Boiled/baked or mashed potatoes |  |  | 0.33 |  |  |
| Fried potatoes | 0.22 |  |  |  |  |
| Rice or pasta | 0.36 |  |  |  |  |
| Vegetables |  | 0.36 |  |  |  |
| Root vegetables (onion, carrot, rutabaga) |  | 0.37 |  |  |  |
| Fruit |  | 0.35 |  |  |  |
| Berries |  |  |  |  |  |
| Dried fruit or fruit and nut mix |  |  |  |  |  |
| Dessert or cakes |  |  |  |  | 0.39 |
| Chocolate or candy | 0.25 |  |  |  | 0.21 |
| Salty snacks | 0.32 |  |  |  |  |
| Nuts or peanut butter |  |  |  |  |  |
| Sauces, sauce butter/margarine melted, or creamy dressing |  |  | 0.28 |  |  |
| Salad dressing like thousand Island, salad dressing oil, mustard, or soy sauce |  |  |  | 0.23 |  |
| Tomato sauce | 0.30 |  |  |  |  |
| Wok with meat/chicken | 0.30 |  |  |  |  |
| Beans/lentils |  |  |  |  |  |
| Stew/soup with fish |  |  |  | 0.28 |  |
| Proportion of variance explained by each dietary pattern, % | 6.2 | 6.0 | 5.8 | 5.1 | 4.5 |
| Cumulative | 27.6 |  |  |  |  |

Abbreviation: PC: Principal Components.

We analysed the associations between five energy-adjusted dietary patterns and periodontitis and number of teeth. We found that the Fruit and vegetables energy-adjusted dietary pattern was associated with increased odds of advanced periodontitis (Supplementary Table 3).

**Supplementary Table 3** Regression models between tertiles of the Fruit and vegetables energy-adjusted dietary pattern scores and periodontitis groups

| Dietary pattern | *Moderate bone loss*  *versus*  *No periodontitis/slow bone loss* | | | *Rapid bone loss*  *versus*  *No periodontitis/slow bone loss* | | | *Rapid bone loss versus*  *Moderate bone loss* | |  |
| --- | --- | --- | --- | --- | --- | --- | --- | --- | --- |
|  | OR  (95% CI) | p-value | p  trend | OR (95%CI) | p-value | p trend | OR  (95% CI) | p-value | p trend |
| *Fruit and vegetables* |  |  |  |  |  |  |  |  |  |
| Tertile 1  (ref.) | 1 |  |  | 1 |  |  | 1 |  |  |
| Tertile 2 | 1.09 (0.81,1.47) | 0.552 | 0.738 | 0.55 (0.31,0.9) | 0.041 | 0.005 | 0.50  (0.28,0.88) | 0.017 | 0.002 |
| Tertile 3 | 1.05 (0.76,1.45) | 0.757 |  | 0.40 (0.20,0.8) | 0.010 |  | 0.38  (0.19,0.76) | 0.006 |  |

Model adjusted for sex, age, education, smoking status, toothbrushing frequency, body mass index, and physical activity.

Abbreviation: OR: odds ratio, CI: confidence interval.

The Refined grain and dessert energy-adjusted dietary pattern was not associated with number of teeth (data not shown).

- 1. **Four retained energy-adjusted dietary patterns**

Since one of the five energy-adjusted dietary patterns, i.e., PC4 that included seafood, eggs, fat fish, mayonnaise, and wine, was difficult to interpret (Supplementary Table 2), we extracted dietary pattern scores when only the first four dietary patterns were retained, which is similar to the method used for the extraction of four unadjusted dietary patterns (Supplementary Figure 2).

**Supplementary Figure 2**

Scree plot for identification of four energy-adjusted dietary patterns (components) by principal component analysis (Bartlett test of sphericity, p<0.001; Kaiser-Meyer-Olkin Measure of Sampling Adequacy=0.775).

Supplementary Table 4 shows four energy-adjusted dietary patterns.

**Supplementary Table 4** Loading matrix (≥|0.20|) and explained variances for the first four energy-adjusted dietary patterns

|  | Fruit and vegetables | Westernised | Meat/fish  and potatoes | Refined grain and dessert |
| --- | --- | --- | --- | --- |
|  | PC1 | PC2 | PC3 | PC4 |
| Refined grains |  |  |  | 0.38 |
| Whole grains 50-100% |  |  |  |  |
| Butter, margarine, or mix of butter, margarine and oil as spread |  |  |  |  |
| Whey cheese |  |  |  | 0.22 |
| White cheese |  |  |  |  |
| Processed meat or paté for sandwiches |  |  |  |  |
| Canned/smoked fish, cod roe from a toothpaste-like tube or shrimp/crab for sandwiches |  |  | 0.23 |  |
| Sweet spreads or sweeteners |  |  |  | 0.37 |
| Mayonnaise salads or mayonnaise as spread/dressing |  |  |  |  |
| Regular milk/soured milk or low-fat milk |  |  |  |  |
| Natural/flavoured yoghurt or milk with probiotics | 0.22 |  |  |  |
| Flavoured chocolate/strawberry milk, milk/cream in coffee/tea or hot chocolate/cocoa |  |  |  |  |
| Water |  |  |  |  |
| Juice |  |  |  |  |
| Sugary drinks (ice tea, soft drinks or fruit/berry drinks) |  |  |  |  |
| Artificially sweetened drinks (ice tea, soft drinks or fruit/berry drinks) and non-alcoholic beer |  | 0.25 |  |  |
| Wine |  |  |  | -0.23 |
| Alcoholic beverages except wine |  |  |  |  |
| Tea |  |  |  |  |
| Coffee | 0.26 |  |  |  |
| Sausage or bacon |  |  | 0.29 |  |
| Processed meat dishes or fast food |  | 0.29 |  |  |
| Red meat |  |  | 0.33 |  |
| Stew meat and chicken |  |  | 0.25 |  |
| Chicken |  | 0.28 |  |  |
| Processed fish |  |  | 0.26 |  |
| Lean fish |  |  | 0.32 |  |
| Fatty fish |  |  |  |  |
| Shrimp/crab or seafood wok |  |  |  | -0.21 |
| Vegetarian dish or soup vegetable |  |  |  |  |
| Eggs and eggs dishes |  |  |  |  |
| Boiled/baked or mashed potatoes |  |  | 0.29 |  |
| Fried potatoes |  | 0.26 |  |  |
| Rice or pasta |  | 0.33 |  |  |
| Vegetables | 0.31 |  |  |  |
| Root vegetables (onion, carrot, rutabaga) | 0.26 |  |  |  |
| Fruit | 0.31 |  |  |  |
| Berries | 0.28 |  |  |  |
| Dried fruit or fruit and nut mix | 0.27 |  |  |  |
| Dessert or cakes |  |  |  | 0.35 |
| Chocolate or candy |  | 0.27 |  |  |
| Salty snacks |  | 0.32 |  |  |
| Nuts or peanut butter | 0.24 |  |  |  |
| Sauces, sauce butter/margarine melted, or creamy dressing |  |  | 0.28 |  |
| Salad dressing like thousand Island, salad dressing oil, mustard or soy sauce |  | 0.21 |  |  |
| Tomato sauce |  | 0.32 |  |  |
| Wok with meat/chicken |  | 0.26 |  |  |
| Beans/lentils |  |  |  |  |
| Stew/soup with fish |  |  |  |  |
| Proportion of variance explained by each dietary pattern, % | 7.4 | 6.4 | 5.8 | 4.9 |
| Cumulative | 24.5 |  |  |  |

Abbreviation: PC: Principal Components.

We analysed the associations between four energy-adjusted dietary patterns and periodontitis. We found a significant negative association between the Fruit and vegetables energy-adjusted dietary pattern and advanced periodontitis (Supplementary Table 5).

**Supplementary Table 5** Regression models between tertiles of the Fruit and vegetables energy-adjusted dietary pattern scores and periodontitis groups

| Dietary pattern | *Moderate bone loss*  *versus*  *No periodontitis/slow bone loss* | | | *Rapid bone loss*  *versus*  *No periodontitis/slow bone loss* | | | *Rapid bone loss versus*  *Moderate bone loss* | |  |
| --- | --- | --- | --- | --- | --- | --- | --- | --- | --- |
|  | OR  (95% CI) | p-value | p trend | OR  (95% CI) | p-value | p trend | OR  (95% CI) | p-value | p trend |
| *Fruit and vegetables* |  |  |  |  |  |  |  |  |  |
| Tertile 1 (ref.) | 1 |  |  | 1 |  |  | 1 |  |  |
| Tertile 2 | 1.00 (0.74,1.35) | 0.983 | 0.567 | 0.58 (0.33,1.02) | 0.056 | 0.006 | 0.57 (0.33,1.00) | 0.051 | 0.011 |
| Tertile 3 | 0.91 (0.65,1.26) | 0.552 |  | 0.39 (0.19,0.80) | 0.010 |  | 0.43  (0.21,0.88) | 0.020 |  |

Model adjusted for sex, age, education, smoking status, toothbrushing frequency, body mass index, and physical activity.

Abbreviation: OR: odds ratio, CI: confidence interval.

We analysed associations between four energy-adjusted dietary patterns and number of teeth. We found that the Refined grain and dessert energy-adjusted dietary pattern was associated with increased odds of having ≤19 teeth (Supplementary Table 6).

**Supplementary Table 6** Regression models between tertiles of the Refined grain and dessert energy-adjusted dietary pattern scores and number of teeth categories

| Dietary pattern | OR  (95% CI) | p-value | p  trend | OR  (95% CI) | p-value | p  trend | OR  (95% CI) | p-value | p  trend |
| --- | --- | --- | --- | --- | --- | --- | --- | --- | --- |
|  |  |  |  |  |  |  |  |  |  |
| *Refined grain and dessert* | *≤19 teeth versus*  *25-28 teeth* | | | *≤19 teeth versus*  *20-24 teeth* | | | *20-24 teeth versus*  *25-28 teeth* | | |
| Tertile 1 (ref.) | 1 |  |  | 1 |  |  | 1 |  |  |
| Tertile 2 | 1.23 (0.64,2.37) | 0.530 | 0.007 | 0.83  (0.42,1.63) | 0.585 | 0.116 | 1.49  (1.06,2.08) | 0.020 | 0.040 |
| Tertile 3 | 2.28  (1.22,4.26) | 0.010 |  | 1.62  (0.85,3.09) | 0.144 |  | 1.41  (0.99,2.00) | 0.055 |  |

Model adjusted for sex, age, education, smoking status, toothbrushing frequency, body mass index, and physical activity.

Abbreviation: OR: odds ratio, CI: confidence interval.

References:

1. Willett W (2013) Nutritional Epidemiology: OUP USA.

2. Willett WC, Howe GR Kushi LH (1997) Adjustment for total energy intake in epidemiologic studies. Am J Clin Nutr 65, 1220S-1228S; discussion 1229S-1231S.
